# Factors associated with youth opposition or support for cannabis legalization in Lebanon

**DOI:** 10.1186/s42238-026-00426-8

**Published:** 2026-04-18

**Authors:** Joseph El‑Khoury, Andre Slim, Michele Cherro, Lilian A. Ghandour

**Affiliations:** 1 Department of Psychiatry, United Arab Emirates University, Al Ain, United Arab Emirates; 2 Department of Epidemiology and Biostatistics, Faculty of Health Sciences, American University of Beirut, P.O. Box 11‑0236, Beirut, Lebanon; 3 Department of Psychiatry, Massachusetts General Hospital, Boston, MA USA

**Keywords:** Legislation, Youth, Lebanon, Medicinal Cannabis, Recreational Cannabis.

## Abstract

**Background:**

In 2020, Lebanon initiated the legalization process for the cultivation of cannabis for medical and industrial purposes only. This paper aims to examine the factors associated with youth attitude towards the legalization of medicinal and recreational cannabis in Lebanon.

**Methods:**

In early 2020, a total of 1,230 young adults aged 18 to 24 participated in an anonymous online survey. The main outcome was the support/opposition of medicinal and recreational cannabis legalization in Lebanon.

**Results:**

Participants who were male, non-students, working, and perceived themselves to be relatively well-off were more likely to be in the supportive rather than oppositional group for attitude towards medicinal (*p-*value < 0.0001). Participants who were also more likely to be supportive of legalization (rather than opposed) were: (1) participants who correctly answered the question regarding the purpose of the legislative change in Lebanon; (2) young adults who perceived cannabis as not harmful; (3) young adults who reported ever using cannabis in combination with alcohol or other drugs; and (4) students who did not think that legalization of cannabis for medicinal use or recreational use would affect their use of other illegal drugs.

**Conclusion:**

Young adults surveyed were generally supportive of cannabis legalization coupled with generally good awareness of the potential risks and harms. From an equity perspective, legalization could create disproportionate harms in different groups of young adults, and therefore any legislative changes should be accompanied by implementing robust public health strategies to address knowledge gaps and mitigate risks in young adults.

## Introduction

Cannabis consumption is a global concern. In 2022, the United Nations Office on Drugs and Crimes (UNODC 2024) estimated that around 228 million people (4.4% of the population aged 15 to 64) use cannabis, signifying a 28% increase since 2002; the Americas and Asia account for over 34% and 27% of the users respectively (UNODC 2024). Still, by January 2024, Canada, Uruguay, and 27 jurisdictions in the United States had legalized the production and sale of cannabis for recreational use, with diverse legislative approaches emerging in other parts of the world (Government 2024, National 2024, Washington 2024). Legalization appears to have intensified harmful cannabis consumption using cannabis products containing high levels of THC (Health 2024, UNODC 2022, UNODC 2024). Such regular cannabis consumption has been linked to an increase in hospitalizations for cannabis use disorders, as well as a growing prevalence of psychiatric conditions and suicide attempts, particularly among young adults in Canada and the U.S (Myran et al. 2023, Wang et al. 2018, Hall et al. 2018, Callaghan et al. 2023).

Studies that have explored public perceptions of cannabis legalization have established that personal experience with cannabis is important in shaping individual perception of its benefits and risks, thereby influencing their stance on legalization. Beyond this, those who perceive cannabis as less risky tend to support legalization, even if they do not personally use the substance (Cohn et al. 2017, Palali and Ours 2017, Elder and Greene 2019, Ellis et al. 2019). Much of the literature on this topic comes from the U.S. and Canada. There is a notable lack of research in the global south, particularly in the Middle East region where cannabis legislation remains broadly strict and punitive. Lebanon is no exception. It is considered one of the leading global producers of cannabis, both for export and domestic use (Afsahi and Darwich 2016). The country ranks as the second largest cannabis producer in the Near and Middle East/Southwest Asia, following Afghanistan (UNODC 2024). Evidence of prevalent local consumption of cannabis products in the general population is backed by official reports and anecdotal public information. A more recent review suggested a lifetime prevalence of 32% in university students (Sabalbal et al. 2025). In 2020, with economic benefits in mind, the Lebanese government passed a law permitting the cultivation of medicinal cannabis with less than 1% THC. This legislation that primarily targets the export market, lacks details on how medicinal cannabis will be made accessible to the public and does not address the legal status of recreational cannabis (Sabalbal et al. 2025).

Globally, cannabis use is generally most prevalent among those aged 18 to 24. Experimentation and initiation often occur during this developmental stage (UNODC 2024, Cohn et al. 2017, El-Khoury et al. 2022). This trend is mirrored in Lebanon through healthcare utilization records and law enforcement statistics. This demographic subgroup is distinctly situated to both impact and be impacted by policy modifications, societal norms, and public discussions regarding cannabis legalization. Consequently, comprehending their attitudes provides valuable insight for customizing harm reduction and prevention strategies, as well as for informing adaptive public health and legislative initiatives.

In a recent publication the authors highlighted a complex interplay between Lebanese youth using cannabis recreationally or for both recreational and medicinal purposes (SAMHSA 2022). Noticeably, dual-motive users—or young adults who reported using cannabis for both recreational and therapeutic purposes—exhibited substantial differences from recreational-only users across multiple domains; they specifically indicated greater usage, a greater tendency to view cannabis as non-harmful, and a heightened likelihood of asserting that legalization—either medicinal or recreational—would affect their future consumption. These findings indicate that this subset of cannabis users may be especially susceptible to the dangers associated with the heightened availability and normalization of cannabis, warranting greater scrutiny in policy formulation and public health initiatives. Building on the same data, this paper aims to examine the factors associated with young adults’ attitude towards the legalization of medicinal and recreational cannabis in Lebanon. Such findings can have both policy and public health implications. Understanding the attitudes of young adults can help tailor prevention and harm reduction interventions to address specific concerns and misconceptions. Understanding the socio-demographic profile of young adults opposed to/pro legalization can also address issues of equity; it is important to consider whether legalization policies may create disproportionate harms in different groups of young adults. Therefore, examining factors associated with attitudes towards legalization behaviours in Lebanon is crucial for establishing evidence-based cannabis laws that take geopolitics, socioeconomic variables, public safety, and health outcomes into account.

## Methods

### Sample recruitment and data collection

An anonymous online survey was launched in January 2021 using LimeSurvey after having received approval from the Institutional Review Board (IRB) at the American University of Beirut in November 2020 [protocol # SBS-2020-0421]. The anonymous online survey method was deemed most suitable given the illegality of cannabis use in Lebanon, in addition to the period of lockdown due to COVID-19, reinforcing the need for an online convenience sampling method. While responses to cannabis use during the preceding 12 months (2020) might have been influenced by the pandemic and related restrictions, no conclusive evidence has confirmed such an effect.

Young adults aged 18–24 residing in Lebanon were invited to participate. The survey link was shared through various social media platforms, including Facebook, Instagram, Twitter (X), and WhatsApp, via personal and professional networks. The invitation text provided essential information about the study’s purpose, inclusion criteria, and assurances of anonymity and data use transparency. Once the survey link was clicked, participants were directed to an online consent form before proceeding to the survey. By March 2021, 1,230 young adults had consented to and completed the survey.

### Data collection

The survey questionnaire included questions on sociodemographics, and covered themes including: cannabis consumption patterns, attitudes toward recreational and medicinal cannabis legalization, perceptions of cannabis-related harms, and the use of other drugs (Ghandour et al. 2024). The survey was initially developed in English by the investigators, building upon the previous surveys and incorporating additional questions relevant to the Lebanese context (Moeller and Woods 2015). The questionnaire was then translated to Arabic and made available to the respondents in the language of their preference (English or Arabic).

### Outcome

Participants were asked about their attitude towards the legalization of medicinal cannabis and recreational cannabis in Lebanon in two separate questions (“*How would you describe your current attitude towards legalization of [medicinal/recreational] cannabis in Lebanon?”).* The responses were categorized into oppositional (against legalization), reserved/neutral, and supportive (in favour of legalization).

### Associated factors

We examined a number of sociodemographics, including age, gender, nationality, main country of residence, student status (yes/no), highest degree, work status, living arrangement (with family or not), and perceived socioeconomic status. 

*Knowledge*-related questions enquired about the latest legislative changes that legalize the cultivation of medicinal cannabis in Lebanon; the question asked participants about the extent to which they were aware of the specifics of the new law. The question posed was:

*“On April 21*,* 2020*,* the Lebanese parliament passed the law legalizing the cultivation of cannabis for medicinal use. Based on what you know and/or have read*,* the new law would (choose only one)*,*”*

and the participants would have to choose the correct answer. Based on their responses, participants were categorized as providing a correct/incorrect answer.

We also included questions that assessed participants’ *perception of harm* from cannabis use if used only once or twice, occasionally, regularly, or daily (each a separate question); responses were Likert responses [not harmful at all, not harmful, somewhat harmful, harmful, very harmful, and don’t know/prefer not to answer]. 

### Statistical analysis

Data was analysed using Stata version 18. Pearson’s Chi-Square was used for the bivariate analyses of categorical data (Tables 1, 2, 3 and 4). In Table 5, multiple binary logistic regression models were run for models 1–3; binary outcomes were generated for both medicinal and recreational cannabis legalization (1 = supportive, 0 = opposing), with neutral/reserved responses and missing data excluded from this particular analyses.

**Table 1:** Sociodemographic Characteristics among Opponents and Proponents of Recreational and Medicinal Cannabis Users

	Attitude towards medicinal cannabis legislation *n *(%)				Attitude towards recreational cannabis legislation *n *(%)			
	Supportive	Neutral/Reserved	Oppositional	*p*-value	Supportive	Neutral/Reserved	Oppositional	*p*-value
Age				0.11				0.41
18–20	213(29.79)	113(35.53)	44(36.63)		136(30.84)	124(31.00)	105(35.12)	
21–24	502(70.21)	205(64.47)	77(63.64)		305(69.16)	276(69.00)	194(64.88)	
Gender				**< 0.001**				**< 0.001**
Female	215(30.54)	167(53.18)	74(61.67)		103(23.68)	176(45.01)	167(56.04)	
Male	489(69.46)	147(46.82)	46(38.33)		332(76.32)	215(54.99)	131(43.96)	
Nationality				0.77				0.91
Lebanese	688(98.29)	298(98.68)	110(99.10)		426(98.61)	386(98.47)	272(98.19)	
Non-Lebanese	12(1.71)	4(1.32)	1(0.90)		6(1.39)	6(1.53)	5(1.81)	
Main residence				0.79				0.88
Lebanon	648(92.44)	294(93.63)	109(93.16)		403(93.29)	364(92.39)	271(92.81)	
Other country	53(7.56)	20(6.37)	8(6.84)		29(6.71)	30(7.61)	21(7.19)	
Student status				**< 0.001**				**< 0.001**
Full-/Part-time	490(70.40)	262(82.91)	95(83.33)		287(67.21)	309(78.23)	240(82.19)	
Not a student	206(29.60)	54(17.09)	19(16.67)		140(32.79)	86(21.77)	52(17.81)	
Highest degree				0.21				0.36
Secondary/High school	140(20.26)	72(23.00)	32(27.83)		92(21.55)	78(20.16)	70(24.05)	
Some university education	156(22.58)	58(18.53)	29(25.22)		93(21.78)	89(23.00)	60(20.62)	
Bachelor	315(45.59)	140(44.73)	44(38.26)		198(46.37)	175(45.22)	117(40.21)	
Masters/MD/PhD	80(11.58)	43(13.74)	10(8.70)		44(10.30)	45(11.63)	44(15.12)	
Work status				**0.004**				**< 0.001**
Working	252(36.10)	84(27.36)	28(24.56)		169(39.12)	119(30.75)	72(25.09)	
Not working	446(63.90)	223(72.64)	86(75.44)		263(60.88)	268(69.25)	215(74.91)	
Living arrangements				0.11				0.001
With family	616(86.88)	288(91.14)	108(90.76)		367(83.98)	360(90.68)	273(92.23)	
Others	93(13.12)	28(8.86)	11(9.24)		70(16.02)	37(9.32)	23(7.77)	
Perceived SES				**0.001**				**0.02**
Little/Lot poorer	114(16.43)	29(9.45)	19(16.81)		74(17.05)	48(12.53)	37(13.03)	
Same	333(47.98)	181(58.96)	67(59.29)		200(46.08)	210(54.83)	165(58.10)	
Little/Lot richer	247(35.59)	97(31.60)	27(23.89)		160(36.87)	125(32.64)	82(28.87)	

**Table 2:** Overview of the Attitudes towards Cannabis among Opponents and Proponents of Recreational/Medicinal Cannabis Users

	Attitude towards medicinal cannabis legislation *n* (%)			Attitude towards recreational cannabis legislation *n* (%)		
	Supportive	Neutral/Reserved	Oppositional	Supportive	Neutral/Reserved	Oppositional
Extent cannabis use harmful if tried only once or twice^1^						
Very Harmful	9(1.28)	17(5.54)	32(26.89)	2(0.46)	11(2.81)	47(16.10)
Harmful	5(0.71)	28(9.12)	19(15.97)	1(0.23)	9(2.30)	41(14.04)
Somewhat harmful	87(12.34)	84(27.36)	27(22.69)	46(10.62)	78(19.95)	70(23.97)
Not Harmful	191(27.09)	94(30.62)	15(12.61)	97(22.40)	123(31.46)	74(25.34)
Not Harmful at all	402(57.02)	66(21.50)	9(7.56)	283(65.36)	159(40.66)	31(10.62)
I don’t know	11(1.56)	18(5.86)	17(14.29)	4(0.92)	11(2.81)	29(9.93)
Extent cannabis use harmful if used once in a while or occasionally^1^						
Very Harmful	6(0.85)	13(4.28)	28(24.14)	2(0.46)	4(1.03)	42(14.63)
Harmful	10(1.42)	33(10.86)	25(21.55)	0(0)	19(4.87)	47(16.38)
Somewhat harmful	57(8.07)	84(27.63)	29(25.00)	19(4.37)	57(14.62)	92(32.06)
Not Harmful	267(37.82)	129(42.43)	13(11.21)	143(32.87)	196(50.26)	63(21.95)
Not Harmful at all	352(49.86)	31(10.20)	5(3.45)	264(60.69)	104(26.667)	19(6.62)
I don’t know	14(1.98)	14(4.61)	17(14.66)	7(1.61)	10(2.56)	24(8.36)
Extent cannabis use harmful if used regularly (once a week or less)^1^						
Very Harmful	22(3.13)	41(13.44)	41(34.75)	2(0.46)	20(5.13)	81(28.03)
Harmful	47(6.69)	92(30.16)	32(27.12)	9(2.08)	60(15.38)	99(34.26)
Somewhat harmful	182(25.89)	85(27.87)	19(16.10)	96(22.22)	135(34.62)	51(17.65)
Not Harmful	265(37.70)	53(17.38)	6(5.08)	175(40.51)	120(30.77)	25(8.65)
Not Harmful at all	170(24.18)	13(4.26)	2(1.69)	141(32.64)	37(9.49)	7(2.42)
I don’t know	17(2.42)	21(6.89)	18(15.25)	9(2.08)	18(4.62)	26(9.00)
Extent cannabis use is harmful if used daily^1^						
Very Harmful	115(16.29)	132(43.56)	81(68.64)	33(7.59)	99(25.45)	191(66.09)
Harmful	152(21.53)	91(30.03)	17(14.41)	75(17.24)	132(33.93)	53(18.34)
Somewhat harmful	270(38.24)56	52(17.16)	7(5.93)	195(44.83)	112(28.79)	19(6.57)
Not Harmful	91(12.89)	8(2.64)	2(1.69)	74(17.01)	19(4.88)	5(1.73)
Not Harmful at all	51(7.22)	0(0)	0(0)	42(9.66)	7(1.80)	2(0.69)
I don’t know	27(3.82)	20(6.60)	11(9.32)	16(3.68)	20(5.14)	19(6.57)

^1^ *P*-value < 0.001

**Table 3:** Behaviors Associations with Support and Opposition of Cannabis Legislation

	Attitude towards medicinal cannabis legislation *n* (%)			Attitude towards recreational cannabis legislation *n* (%)		
	Supportive	Neutral/Reserved	Oppositional	Supportive	Neutral/Reserved	Oppositional
Ever used cannabis in combination with alcohol or other drugs^1^	398(57.51)	52(16.77)	11(9.32)	292(68.87)	137(35.31)	30(10.17)
Ever operated a vehicle within two hours of using cannabis^1^	315(44.06)	45(14.15)	9(7.44)	243(55.10)	98(24.50)	28(9.36)
Ever been a passenger in a motorized vehicle with someone who had used cannabis within the preceding two hours?^1^	424(59.30)	95(29.87)	19(15.70)	307(69.61)	171(42.75)	57(19.06)

^1^ *P*-value < 0.001

**Table 4:** Attitudes towards the New Cannabis Law among Opponents and Proponents of Recreational/Medicinal Cannabis Users

	Attitude towards medicinal cannabis legislation *n* (%)			Attitude towards recreational cannabis legislation *n* (%)		
	Supportive	Neutral/Reserved	Oppositional	Supportive	Neutral/Reserved	Oppositional
Current legalization of cannabis in Lebanon will affect participant cannabis consumption^1^						
Yes	136(19.71)	52(17.11)	5(4.46)	80(18.52)	90(23.81)	20(7.07)
No	509(73.77)	229(75.33)	98(87.50)	330(76.39)	253(66.93)	246(86.93)
PNA	45(6.52)	23(7.57)	9(8.04)	22(5.09)	35(9.26)	17(6.01)
Legalization of cannabis for medicinal use only in Lebanon would encourage participant attempt to try other illegal drugs^1^						
Yes	17(2.39)	14(4.43)	14(11.76)	9(2.05)	14(3.54)	18(6.04)
No	652(91.83)	267(84.49)	94(78.99)	403(92.01)	350(88.38)	254(85.23)
Unsure	41(5.77)	35(11.08)	11(9.24)	26(5.94)	32(8.08)	26(8.72)
Legalization of cannabis for recreational use only in Lebanon would encourage participant to try other illegal drugs^1^						
Yes	20(2.82)	21(6.67)	18(15.00)	12(2.75)	13(3.28)	32(10.74)
No	640(90.27)	260(82.54)	91(75.83)	395(90.39)	343(86.62)	245(82.21)
Unsure	49(6.91)	34(10.79)	11(9.17)	30(6.86)	40(10.10)	21(7.05)

^1^ *P*-value < 0.001

**Table 5:** Multiple regression models

Predictor (ref. category)	Outcome1: Pro Legalization for Medicinal Use OR (95% CI)	*p*-value	Outcome 2: Pro Legalization for Recreational Use OR (95% CI)	*p*-value
Model 1: Sociodemographics				
Sex (Male vs. Female)	**3.59 (2.60–4.98)**	**< 0.001**	**3.85 (2.85–5.22)**	**< 0.001**
Student status (Non-student vs. Student)	**2.04 (1.30–3.16)**	**0.002**	**1.87 (1.28–2.92)**	**0.001**
Job status (Non-working vs. Working)	0.92 (0.63–1.35)	0.667	**0.71 (0.51–0.99)**	**0.045**
Self-perceived SES				
Same as others vs. Richer	0.75 (0.45–1.22)	0.263	0.79 (0.51–1.23)	0.302
Little/lot richer vs. Richer	1.10 (0.63–1.91)	0.737	1.26 (0.79–2.01)	0.325
Model 2: Risky Behaviors				
Ever used cannabis with alcohol/other drugs *(ref = No)*	**2.27 (1.15–4.47)**	**0.018**	**2.38 (1.50–4.00)**	**< 0.001**
Ever operated a vehicle within 2 h of use*(ref = No)*	**2.55 (1.21–5.36)**	**0.013**	1.50 (0.91–2.63)	0.115
Ever been passenger with impaired driver *(ref = No)*	1.32 (0.71–2.67)	0.448	1.68 (0.97–3.10)	0.064
Model 3: Attitude towards legalization				
Law legalizing cannabis would affect own use (No vs. Yes)	**0.27 (0.15–0.47)**	**< 0.001**	**0.49 (0.30–0.74)**	**0.001**
Law legalizing cannabis would encourage trying other drugs†				
No (vs. yes)	**3.37 (1.45–7.84)**	**0.005**	**3.12 (1.62–6.10)**	**0.001**
Unsure (vs. yes)	1.40 (0.52–3.76)	0.502	**2.52 (1.11–5.71)**	**0.027**
Model 4: Level of harm perception				
Harm level (0–4)⸸	**0.27 (0.22–0.28)**	**< 0.001**	**0.25 (0.20–0.31)**	**< 0.001**

† For medicinal support, the predictor is *“Medicinal legalization would encourage trying other drugs.* For recreational support, the predictor is *“Recreational legalization would encourage trying other drugs*

⸸ Ordinal variable (0 = lowest level of perceived harm)

For Table 5, model 4, and to examine the association between perceived harm of cannabis and support for its legalization, we constructed a composite harm perception scale (range 0–4). This scale was derived from four items assessing perceived harmfulness of cannabis at different frequencies of use (once or twice, occasional, regular, daily). Each item was dichotomized (0 = not harmful, 1 = harmful), and responses were combined hierarchically such that higher scores represented endorsement of harmfulness at more levels of use.

Bivariate ordinal logistic regression was performed using harm perception as the independent variable and support for legalization as the dependent variable. The critical alpha level in all analyses was set at 0.05.

## Results

### Sample characteristics

Our sample involved 1230 participants. Those aged 18–20 years constitute 32.36% (*n* = 398) of our sample, while the remaining 67.64% (*n* = 832) were aged 21–24 years. More than half of the sample (*n* = 723, 59.5%) were males compared to 40.4% (*n* = 490) females. The vast majority were Lebanese (98.39%) *n* = 1161) and residing mainly in Lebanon (92.96%, *n* = 1123). In terms of education, most participants are full/part-time students (75.52%, *n* = 907) and have at least a bachelor’s degree (55.74%, *n* = 665). Around 68% (810) reported being unemployed, in addition to 88.76% (082) living with family members. When asked about their perceived socioeconomic status, 14.89% (76) reported being a little/lot poorer, 52.54% (621) reported being the same, and 32.57% (385) reported being a little/lot richer than others.

### Attitude towards cannabis legalization by sociodemographics

Table 1 shows the distribution of attitude towards the legalization of medicinal and recreational cannabis (separately) by sociodemographics. As can be seen, the percentage of males was statistically significantly higher in the supportive than the oppositional groups for both attitude towards medicinal (70% vs. 38%, *p-*value < 0.001) and recreational cannabis legalization (76% vs. 44%, *p-*value < 0.0001). Statistically significant differences are also seen by student status, true for both medicinal and recreational cannabis legalization. When asked about their attitude towards recreational cannabis legalization, 67% of the supportive group were students vs. 82% of the oppositional group (*p-*value < 0.001). This complements the picture painted by working status, whereby a higher percentage of ‘working’ participants were supportive to both medicinal and recreational cannabis legalization. In terms of perceived socioeconomic status, there was a higher percentage of respondents who perceive themselves as little/lot richer in the supportive rather than oppositional group for both medicinal cannabis legalization (36% vs. 24%, *p-*value: 0.001) and recreational cannabis legalization (37% vs. 29%, *p-*value: 0.02). The remaining examined sociodemographics (age, nationality, main residence, highest degree obtained, and living arrangement) were not statistically significantly associated with attitude towards cannabis legislation.

### Knowledge about the new legislative changes

Those who correctly answered the question regarding the new legislation were more prevalent in the supportive rather than oppositional groups for both medicinal cannabis legislation (66% vs. 45%) and recreational cannabis (62% vs. 53%) (*P-value* < 0.001).

### Perception of cannabis harm

Table 2 looks at the distribution of perception of harm towards varying frequencies of cannabis use and attitude towards cannabis legalization, both medicinal cannabis legalization and recreational. Expectedly, the percentage of participants who reported that it would be very harmful if cannabis was tried once or twice, or tried occasionally, or used regularly, or daily was highest in the oppositional group, followed by the neutral/reserved group, and lowest among the supportive group. For example, only 17% of those who supported medicinal cannabis legalization reported that it would be very harmful to use cannabis daily, in contrast to 69% of the group opposed to such legalization. From Table 2, one could deduce that (UNODC 2024) perception of harm was higher in the groups with stricter attitudes towards legalization, and (Government 2024) the percentage of young adults even within the supportive group reporting that cannabis was ‘very harmful’ increased as the frequency of cannabis use increased (1% thought trying was very harmful, compared to 17% thinking daily use was very harmful).

### Risky cannabis-related behaviours

Table 3 shows the association between risky behaviours and attitude towards legalization. The percentage of young adults who reported ever using cannabis in combination with alcohol or other drugs was highest in the supportive groups (58% and 29% in groups supporting medicinal and recreational legalization, respectively) versus 9% and 10% in groups opposing medicinal and recreational legalization, respectively. The same was observed for other behaviours, including ever operating a vehicle within two hours of using cannabis, and ever being a passenger with someone who has. From this data, one could deduce that young adults who engaged in risky behaviours had a more lenient attitude towards cannabis legalization.

### Perceived impact on personal use

Young adults were asked whether the recent legislative changes were going to affect their cannabis consumption, and the percentage who responded positively (% yes) was highest in the supportive group and lowest in the oppositional. When asked whether legalization of cannabis for medicinal use only or legalization of cannabis for recreational use only would affect their use of other illegal drugs, the percentage of young adults who answered positively was lowest in the supportive groups and highest in the oppositional (Table 4). In other words, those who were against the cannabis legalization were more likely to believe that any cannabis legalization would increase the use of other illegal drugs. For example, 3% of those supportive of recreational cannabis use reported that recreational cannabis use would encourage them to try other illegal drugs, in contrast to 11% of those opposed to legalization (Table 4).

Table 5 shows the results of binary logistic regressions predicting support for cannabis legalization for medicinal and recreational purposes. For both medicinal and recreational use, participants who were supportive of their legalization, were more likely to be male [3.59 (2.6–4.98)] for medicinal and 3.85 (2.85–5.22) for recreational] and non-students [2.04 (1.3–3.16) for medicinal and 1.87 (1.28–2.92) for recreational] (Table 5, model 1). Participants who reported using cannabis with alcohol/other drugs were twice as likely to be supportive of legalization (for recreational and/or medicinal purposes) (Table 5, model 2). Young adults who believed that a law legalizing cannabis would not affect their own use were less supportive of legalization. However, those who believed that cannabis legalization would not encourage illicit drug use were more supportive [3.37 (1.45–7.84) for medicinal and 3.12 (1.62–6.10) for recreational].

Our findings further demonstrated a strong inverse association between harm perception and support for cannabis legalization (Table 5, model 4). For medicinal cannabis, each one-unit increase in harm perception was associated with a 73% decrease in the odds of support (OR = 0.27, 95% CI: 0.22–0.28, *p* < 0.001). Similarly, for recreational cannabis, each one-unit increase in harm perception was associated with a 75% decrease in support (OR = 0.25, 95% CI: 0.20–0.31, *p* < 0.001).

## Discussion

In this study from Lebanon, we examine young adults’ attitudes towards the legalization of cannabis for medicinal and/or recreational purposes and describe the socio-demographic and other characteristics differentiating those who oppose from those who support legalization. The significance of the country is in its positioning both as a prominent producer, so far illicitly, of cannabis and a consumer of the substance for recreational purposes. Besides Iran, Lebanon is also one of the few countries with a significant Muslim population and a fragile geopolitical context where this subject has been studied. A review of the attitudes of youth towards substance use in this part of the world suggest a role, amongst others, for religious dogma that may be absent in western societies (Khoury et al. 2019). In our sample around 60% were supportive of recreational cannabis legalization, in line with the percentage of supportive youth in Australia and the US, despite methodological differences (Ghandour et al. 2024, Khoury et al. 2019). We observed important variations, particularly that young adults who were male, non-students, working and of a higher perceived socioeconomic status were the ones most likely to be supportive of cannabis legalization for medicinal and recreational use. The profile of cannabis legalization supporters seems to vary between countries. A survey of young people from Finland suggests that females are more likely to support legalization for medical purposes (Rudy et al. 2021), while an Australian study found older females to be the most liberal on cannabis legalization. These differences could also reflect different gender dynamics influenced by the social, cultural and religious context of Lebanon. The social structure in Lebanon remains highly patriarchal, with males being significantly more likely to voice controversial opinions or act on the fringe of lawful boundaries. This is particularly the case within low SES circles where the influence of tradition and religious conservatism is stronger.

Another interesting finding is the association between wealth perception and support for legalization; young adults in our sample who perceived themselves to be wealthier were more supportive of the legalization of cannabis (both for recreational and medicinal purposes). It is established that individuals with higher socioeconomic status usually experience fewer legal or financial consequences related to cannabis use, paving the way for more liberal attitudes on legalization (Chiu et al. 2022). Additionally, some studies argue that wealthier individuals might be more involved in communities that advocate for reform, influencing their attitudes through more liberal social networks (Hupli et al. 2024). As highlighted by studies on drug policy attitudes, higher socioeconomic groups often have more exposure to discussions around cannabis and its benefits, which can further shape their perceptions more positively (Shover and Humphreys 2019). The intersection between socioeconomic status and perception towards cannabis legislation is multifaceted, involving personal experience, community affluence, and societal trends. This complex relation can vary from one country to another as it integrates the sociocultural and religious context.

Expectedly, the perception of cannabis harm was highest for daily use and lowest for trying once or twice, and this was true regardless of attitude towards legalization. Quite interestingly, the perception of harm was always higher in the oppositional rather than supportive groups (the latter more likely to perceive cannabis as less harmful). This is in line with ongoing social debates about the risks versus the benefits of cannabis, with potential implications for health campaigns aimed at educating the public about safe use practices (Pacula and Smart 2017). From global experience, this debate does not end when cannabis is legalized. Even in countries with progressive cannabis laws, scepticism about its widespread use is prevalent (Stephenson et al. 2023).

In terms of actual consumption, our study revealed some meaningful behavioural trends in cannabis use. Over 40% of respondents reported using cannabis in conjunction with alcohol or other drugs. Nearly one-third reported driving under the influence of cannabis. International experience so far suggests that legalization does not improve this particular risky behaviour. While more research is needed, impaired driving under the influence has been highlighted as a public health issue where cannabis has been legalized (Hall and Weier 2015). Our survey further highlighted that the percentage of young adults driving under the influence of cannabis was highest in those supportive of legalization and lowest in those who were oppositional. A 2022 scoping review found, among other factors, that having a reduced perception of danger was linked to a higher likelihood of driving under the influence of cannabis (DUIC); reduced perception of cannabis harm in our study was indeed linked to more lenient attitudes towards legalization and the latter was linked to a higher likelihood of DUIC (Lopez-Quintero et al. 2011). If indeed easier access to cannabis legally means more young people driving under its influence, there is a need for clear regulations and educational interventions to prevent this and other dangerous behaviours associated with cannabis use. A major challenge for a fragile country such as Lebanon is the weakness of the central state authority and the inability of law enforcement agencies to effectively police a surge in antisocial behaviour involving cannabis directly or indirectly.

There are limitations to the study. First, given the sampling methodology adopted, selection bias cannot be ruled out, and the young adult sample is likely not representative of Lebanon’s young adult population. The survey was released within months of the news of cannabis legalization for therapeutic use, a period that coincided with the COVID-19 pandemic and the national lockdowns. An online survey was the only viable choice, and therefore the convenience sampling adopted to recruit young adults aged 18 to 24 is likely to have resulted in selection bias. In fact, when comparing our estimates to the statistics made available by CAS/ILO, our sample appears to be skewed toward certain subgroups of Lebanese young adults (ILO 2018). In particular, it over-represents males, university students, and Bachelors’ degree holders, while under-representing less-educated or non-student youth. The proportion reporting unemployment (68%) is higher than official youth unemployment estimates (~ 48%), likely reflecting differences in definition and a student-heavy sample. Socioeconomic self-perceptions in our sample also suggest relatively better-off participants than expected in the broader population. Overall, our sample provides insight into digitally connected and educated young Lebanese, and is not fully representative of the national 18–24 cohort. Another limitation is inherent to the cross-sectional nature of the study, which precludes causal inference regarding risky behaviours, perceptions of harm and legalization attitudes. While causality or bidirectionality cannot be established in a cross-sectional design, the observed associations remain valuable in highlighting potential prevention and intervention pathways. These insights, however, should be interpreted with caution, as our sample—being more male, more highly educated, and more student-oriented than the broader Lebanese youth population—reflects a particular youth profile. Accordingly, prevention approaches informed by these findings may be most relevant to similarly profiled subgroups of young adults.

### Summary and recommendations

In summary, we found a high prevalence of supportive attitudes for medicinal and recreational cannabis legalization among young adults in Lebanon, with education, age, and perceived wealth playing significant roles. However, concerns about the harms of cannabis use, particularly in the context of regular use and risky behaviours, highlight the need for comprehensive public health strategies to mitigate risks. By identifying the demographic and socioeconomic factors that shape young adults’ perceptions, researchers and policymakers can collaborate to create informed, evidence-based policies. These efforts can promote safer cannabis use while reducing potential harms associated with its misuse. In doing so, public health and educational programs can be more effective, fostering healthier behaviours and informed decision-making among young adults. Based on our findings, we recommend the following evidence-informed recommendations:

#### Target high-risk groups

Prevention efforts must focus on subgroups most likely to support or engage in risky cannabis use. Interventions should include tailored messaging and peer-led initiatives. Special attention should be given to driving under the influence of cannabis (DUIC), as nearly one-third of youth in our study reported this behaviour.

#### Strengthen education and awareness campaigns

Any messages related to cannabis and its associated harms must be delivered in a balanced, evidence-based manner and must consider mental health effects and impaired driving. Universities, vocational centers, and digital platforms can serve as primary outreach channels. Educational institutions are encouraged to host debates, seminars, and health fairs to expose students to evidence-based information on risks versus benefits. Digital strategies, including interactive campaigns (quizzes, challenges, short videos) on platforms like Instagram, TikTok, and WhatsApp, can support in debunking common myths (e.g., “cannabis is harmless”) and promote scientifically grounded messages.

#### Conduct further research and monitoring

Given the limitations in representativeness, future studies should aim to gather data from a nationally representative sample of youth residing in Lebanon. Regular monitoring through substance use surveillance systems will further allow prevention programs to adapt dynamically to changing trends and emerging risk patterns.

#### Embed prevention within policy reform

Our findings clearly highlight the need for a clear regulatory framework following any future legalization efforts. Robust safeguards must include minimum age restrictions, advertising limitations, road safety enforcement, as well as a dedicated funding for youth-focused prevention and harm-reduction programs.

#### Foster community-based engagement

While not directly linked to one of our findings, engaging families, youth clubs, civil society organizations, and local community groups to extend prevention messages beyond schools and digital platforms can help frame the issue around shared values such as health, safety, and social responsibility—and ensure that messages are credible, culturally relevant, and well-received by young adults.

This study is one of the very few to explore cannabis usage and its correlation with attitude towards change in legislation outside of the usual developed countries where cannabis legalization has already made leaps forward. The example of Lebanon, a small nation in the grips of a chronic structural instability exemplifies the importance of geopolitical context. Given that it is on the verge of implementing significant drug policy reforms specific to cannabis, understanding the views and actions of its young adults provides essential insights for proactive harm reduction, educational initiatives, and equitable regulatory frameworks. Youth attitudes towards cannabis legalization is a significant factor that can influence individual behaviour, policy decisions, and public health outcomes. More research is needed to fully understand the interface between societal attitudes towards cannabis legalization on a global scale and the effective short- and long-term implications of these reforms. All while taking into consideration the role of social structures, religious ideologies, local culture and health resources.

## Data Availability

The datasets used and/or analysed during the current study are available from the corresponding author on reasonable request.

## Abbreviations

IRB: Institutional Review Board
UNODC: United Nations Office on Drugs and Crimes
